# The COP9 signalosome stabilized MALT1 promotes Non-Small Cell Lung Cancer progression through activation of NF-κB pathway

**DOI:** 10.1007/s10565-024-09888-z

**Published:** 2024-06-12

**Authors:** Yinghui Wang, Xuyi Deng, Jing Xie, Tianhao Lu, Rui Qian, Zhi Guo, Xin Zeng, Jing Liao, Zhenhua Ding, Meijuan Zhou, Xinli Niu

**Affiliations:** 1 Department of Radiation Medicine, Guangdong Provincial Key Laboratory of Tropical Disease Research, NMPA Key Laboratory for Safety Evaluation of Cosmetics, School of Public Health, Southern Medical University, Guangzhou, Guangdong Province China; 2 Jiangmen Central Hospital, Affiliated Jiangmen Hospital of Sun Yat-Sen University, Jiangmen, Guangdong Province China; 3 Department of General Surgery, Nanfang Hospital, Southern Medical University, Guangzhou, Guangdong Province China

**Keywords:** MALT1, COP9 signalosome, Ubiquitination, FBXO3, NF-κB

## Abstract

MALT1 has been implicated as an upstream regulator of NF-κB signaling in immune cells and tumors. This study determined the regulatory mechanisms and biological functions of MALT1 in non-small cell lung cancer (NSCLC). In cell culture and orthotopic xenograft models, MALT1 suppression via gene expression interference or protein activity inhibition significantly impaired malignant phenotypes and enhanced radiation sensitivity of NSCLC cells. CSN5, the core subunit of COP9 signalosome, was firstly verified to stabilize MALT1 via disturbing the interaction with E3 ligase FBXO3. Loss of FBXO3 in NSCLC cells reduced MALT1 ubiquitination and promoted its accumulation, which was reversed by CSN5 interference. An association between CSN5/FBXO3/MALT1 regulatory axis and poor prognosis in NSCLC patients was identified. Our findings revealed the detail mechanism of continuous MALT1 activation in NF-κB signaling, highlighting its significance as predictor and potential therapeutic target in NSCLC.

**Graphical Abstract:**

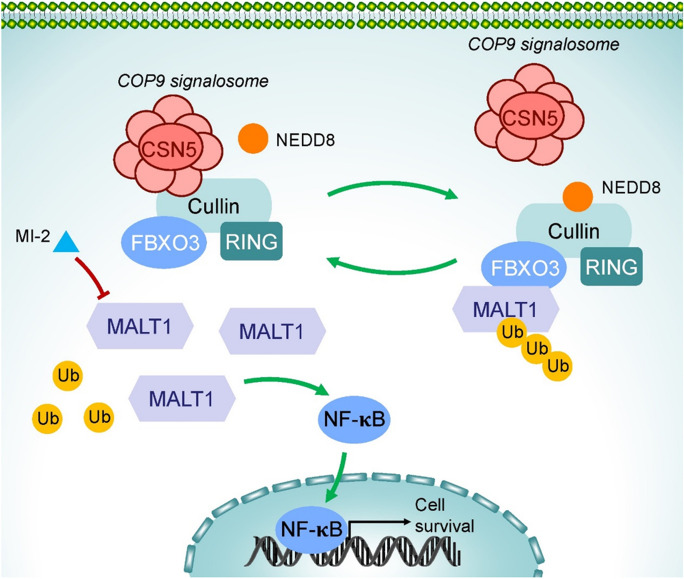

**Supplementary Information:**

The online version contains supplementary material available at 10.1007/s10565-024-09888-z.

## Introduction

Lung cancer has second morbidity and highest mortality of cancer worldwide (Sung et al. 2021). From 2011 to 2017, the 5-year survival rate of patients with non-small cell lung cancer (NSCLC) was only 26% (Siegel et al. 2022). The emerging molecular biotechnologies and development of new drugs have resulted in the application of targeted therapies for ~ 69% of advanced NSCLC patients with specific genomic aberrations; however, the efficiency of these therapies is limited by off-target effects and therapeutic resistance (Hirsch et al. 2017). To date, it remains necessary to determine the molecular mechanisms of NSCLC and identify additional targets to improve the efficacy of therapies.

NF-κB, a rapidly inducible transcription factor, is a valuable pharmaceutical target due to its indispensable role in cancer malignant progression and therapeutic resistance (Taniguchi and Karin 2018; Mortezaee et al. 2019). Several NF-κB inhibitors have been marketed and used as first-line treatment for multiple myeloma (Huang et al. 2014). However, safety and efficiency concerns about the existing drugs drive researchers to search for new targets in cancer treatment (Rasmi et al. 2020; Ramadass et al. 2020). Mucosa-associated lymphoid tissue lymphoma translocation protein 1 (MALT1), upstream of NF-κB pathway, forms CBM complex with CARMA1 and BCL10 to activate IKK complex or functions as a caspase-like protease that promotes prolonged NF-κB activation (Wiesmann et al. 2012). Extra MALT1 copies in MALT lymphoma patients are usually associated with relapse or radiotherapy resistance (Nakamura et al. 2007; Ishikawa et al. 2015). MALT1 has carcinogenic roles in solid tumors, such as colorectal cancer, glioma and breast cancer (Liu et al. 2020; Qian et al. 2022; J. L. Lee et al. 2021). Recently, MALT1 inhibition by small molecules has been verified to significantly impair tumor growth in vivo without side effects, highlighting its potential in targeted therapy (Fontan et al. 2012). However, while previous studies have indicated the essential contribution of MALT1 to NF-κB activation (Rehman and Wang 2009; Ekambaram et al. 2018), the exact underlying mechanisms involved, especially how the oncogene effect is maintained, remain unclear.

COP9 signalosome (CSN) is an evolutionarily conserved protein complex in eukaryotic cells that inactivates all Cullin-RING ubiquitin E3 ligases (CRLs) by removing the activator NEDD8 (Lingaraju et al. 2014). Since CRLs constitute the majority of ubiquitin ligases controlling over 20% of all ubiquitination events, CSN governs the ubiquitylation status of almost every cellular pathway. Several recent researches have suggested CSN has a critical role in NF-κB activation (Dubiel et al. 2020; Harper and Schulman 2021; Schweitzer and Naumann 2010). IκBα was early reported to be deubiquitinated by CSN in cooperation with USP15 or matrix protein 2 (Schweitzer et al. 2007; J. H. Lee et al. 2013). UPS dependent degradation of RelA is inhibited by CSN (Schweitzer and Naumann 2015). However, CSN mediated ubiquitinylated activation of IKK complex facilitates TCR induced NF-κB activation. Several other substrates, such as BCL10, are known to be involved in T-cell signaling (Welteke et al. 2009). Therefore, how CSN precisely regulates NF-κB activation and through which component have yet to be determined, and additional experiments are needed in different contexts.

In this study, we verified that inhibiting MALT1 function suppressed tumor malignancy in NSCLC. Mechanistically, we found that MALT1 recruits CSN, CRL-negative regulator, which reduces MALT1 ubiquitination mediated by E3 ligase FBXO3 and suppresses its degradation. Our study provides insight into the involvement of the stabilized MALT1 “system” in ubiquitin-linking and deneddylation, which contributes to the hyperactivation of MALT1 in NSCLC malignancy and provides a bright intervention target for clinical therapy.

## Materials and methods

### Patient samples and cell lines

In this study, NSCLC samples and adjacent noncancerous tissues were collected from 10 patients. The Ethical Committee of Jiangmen Central Hospital approved the study, and all patients provided informed consent. Four NSCLC cell lines (A549, H460, H1650, and sk-lu-1) and a human normal bronchial epithelial cell line (BEAS-2B) were cultured in Dulbecco’s modified Eagle medium (DMEM, Gibco, Carlsbad, USA). The chemical reagents MG-132 and cycloheximide (CHX) were obtained from SelleckChem (Houston, USA), and MI-2 was obtained from TargetMol (Boston, USA).

### Immunohistochemistry

The NSCLC sections were stained with a MALT1 antibody (Proteintech, 66225–1-Ig, Wuhan, China), and the staining intensity was rated from negative to strongly positive on a scale of 0 ~ 3. A score of 0 ~ 4 corresponds to the percentage of positive cells as no positive cells, less than 10%, 10–50%, 50–80%, and greater than 80%, respectively. The product of these two scores was the final score. The scoring was performed by a pathologist double-blinded to the antigen probe and sample identity.

### Western blotting and co-immunoprecipitation

Protein was extracted in cell lysis buffer (Beyotime, Shanghai, China) containing protease inhibitor (SelleckChem), separated using sodium dodecyl sulfate–polyacrylamide gel electrophoresis (SDS-PAGE), and blotted onto PVDF membranes (Millipore, MA, USA), which incubated with primary antibodies against MALT1 (CST, 2494, Danvers, USA), CSN5 (Proteintech, 27511–1-AP), FBXO3 (Proteintech, 17803–1-AP), p-p65 (Absci, AB11014, OR, USA), Ub (Proteintech, 10201–2-AP), β-actin (Santa Cruz, 47778, CA, USA), and GAPDH (Santa Cruz, 47724). The bound primary antibodies were visualized using Luminata Forte Western HRP substrate (Millipore) after incubation with secondary antibody anti-mouse IgG-HRP or anti-rabbit IgG-HRP (Invitrogen, CA, USA). Co-immunoprecipitation (Co-IP) was conducted by using protein A/G magnetic beads (Bimake, Houston, USA) following the manufacturer’s instructions.

### Reverse transcription and qPCR

TRIzol (Invitrogen) was utilized to extract total RNA following instructions of manufacturer. Evo M-MLV RT Kit (Accurate Biology, Hunan, China) and PerfectStart Green qPCR SuperMix (TransGen Biotech, Beijing, China) were utilized for reverse transcription and qPCR, respectively. GAPDH was used as an internal reference gene and qPCR was conducted on LightCycler 96 Detection System (Roche, Basel, Switzerland). The primer sequences are shown in Table S1.

### Cell proliferation assay

A Cell Counting Kit-8 (CCK-8, Dojindo, Kumamoto, Japan) measured cell viability. Cells were transfected with siRNA (RIBOBIO, Guangzhou, China) or plasmid (Hunan Fenghui Biotechnology Co., Hunan, China), and the siRNA sequences are shown in Table S1. Optical density (OD) values were detected at 0, 24, 48, and 72 h after transfection. Cells were seeded into 6-well plates (1,000 cells/well) for colony formation assay and incubated for 2 weeks. Then cells were fixed in 3.7% formaldehyde for 30 min and stained with 0.1% crystal violet. ImageJ was used to count colonies (≥ 50 cells).

For radiation sensitivity assay, cells were irradiated at varying doses (0, 2, 4, 6, 8 Gy) using the Faxitron MultiRad 225 X-ray machine (2 Gy/min, MA, USA). The surviving fraction was calculated as follows: colonies counted/ (cells plated × plating efficiency), where plating efficiency calculated as follows: (unirradiated colony counts/cells seeded for unirradiated controls) × 100%.

### Migration and invasion assays

After transfection of siRNA or plasmid, the migration of cells was evaluated by seeding them into 8 μm cell culture inserts of 24-well plates (Millipore). Matrigel-coated chambers (Corning, NY, USA) were utilized for cell invasion assay. After a culture period of 14-16 h, the cells in upper chambers were fixed via 3.7% formaldehyde and permeabilized with methanol. Then, 0.1% crystal violet stained the upper chambers and the images were obtained by microscope (ZEISS, Oberkochen, Germany).

### Dual-luciferase reporter assay

Cells were co-transfected with pNFκB-luc plasmid (Beyotime) and siRNA or plasmid; after 48 h incubation, luciferase intensity was measured by a dual-luciferase reporter assay kit (Beyotime) following the manufacturer’s instructions.

### LC–MS analysis

The immunoprecipitated protein mix was subjected to SDS-PAGE and digested by In-Gel Tryptic Digestion Kit (Thermo Scientific, MA, USA). The resulting digest was lyophilized, dissolved in a 1% formic acid solution, and subsequently analyzed using a Thermo Fisher Scientific Orbitrap Fusion LC–MS in positive ion mode. The data were searched using Proteome Discoverer software (Thermo Scientific).

### Immunofluorescence assay

A 12-well plate was seeded with cells on glass coverslips for 24 h. Following treatment, the cells were fixed and permeabilized with 4% paraformaldehyde and 0.5% NP-40, respectively. Then, cells were blocked with 3% BSA and incubated with primary antibodies and specific fluorescence-conjugated secondary antibodies. The primary antibodies were used as follows: Rad51 (Abcam, ab133534, Cambridge, UK), phosphorylated DNA-PKcs (Abcam, ab124918), and γ-H2AX (Abcam, ab26350). DAPI was used to stain the nuclei. The ZEISS confocal laser scanning microscope was utilized to acquire images.

### Animals

Athymic nude mice (male, 6–8 weeks of age) were obtained from Guangdong Medical Laboratory Animal Center. Southern Medical University Animal Care and Use Committee approved all animal experiments. For the subcutaneous xenograft model, siNC- or siMALT1- transfected H460 cells (5 × 10^6^) were subcutaneously injected into the two flanks of nude mice. SiNC and siMALT1 were injected into the corresponding tumors every 2 days, beginning 7 days after cell injection. Tumor volume was calculated as longest diameter × (shortest diameter)^2^/2 and measured every 2 days.

For the orthotopic lung cancer model, mice were anesthetized and positioned in the right lateral decubitus. Luciferase-tagged A549 cells (4 × 10^6^) were directly injected into the left lateral thorax (3-5 mm depth) using an insulin syringe. Tumor growth was monitored every 7 days using the noninvasive bioluminescence imaging system (Bruker FX Pro, MA, USA). Mice were randomized into two groups after 1 week injection and treated daily with DMSO or MI-2 (25 mg/kg) via intraperitoneal injection.

### Statistical analysis

The experimental results were expressed as mean ± standard deviation (SD). One-way ANOVA was performed to determine the significance of difference between two groups using SPSS 22.0 software. P-value less than 0.05 were considered significant.

## Results

### MALT1 inhibition impairs NSCLC cell proliferation

**Fig. 1:**
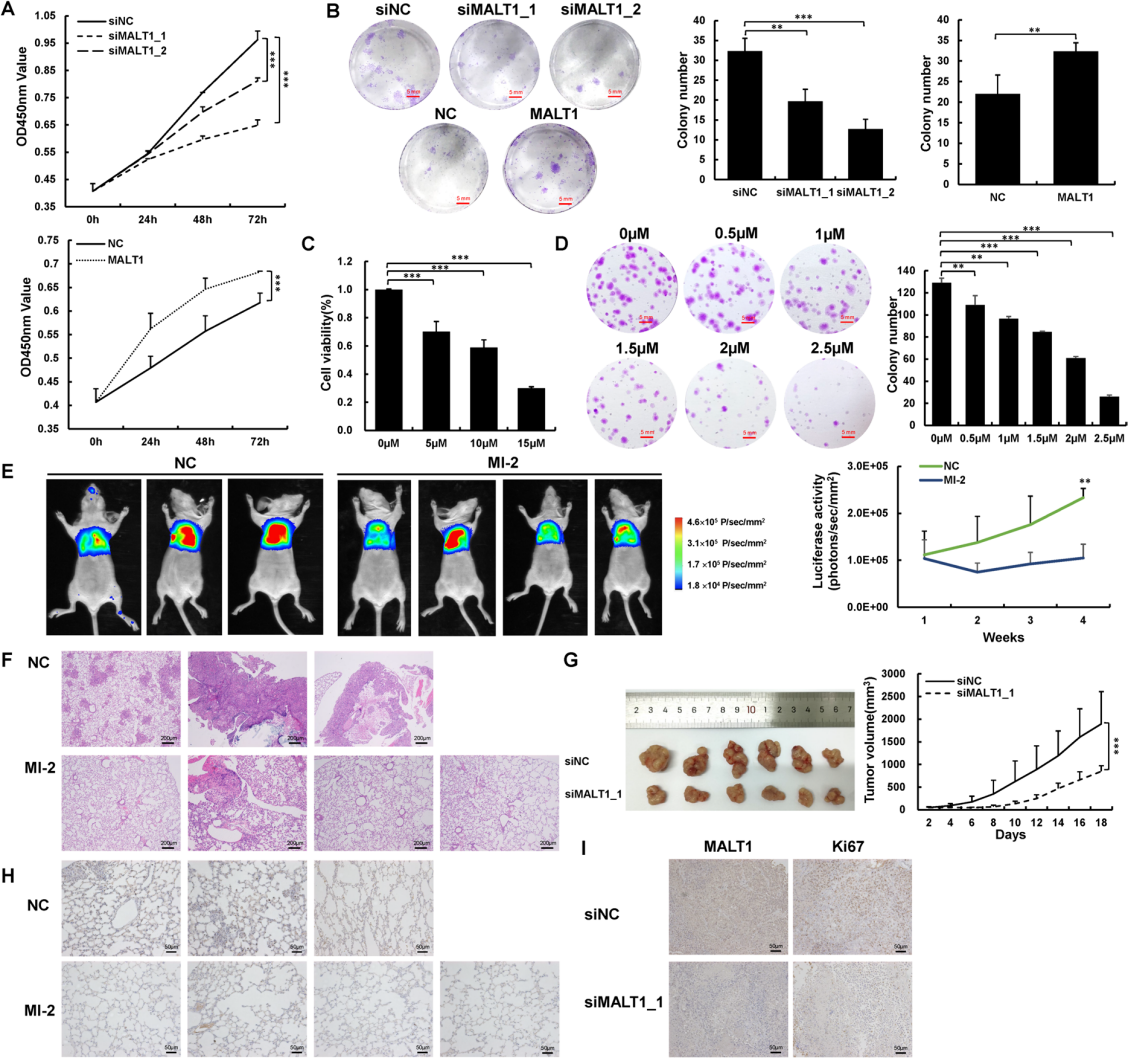
MALT1 inhibition impaired proliferation of NSCLC cells. **A** Cell proliferation of A549 cells was determined by CCK-8 assays at 24, 48, 72 h after transfection, as described. **B** Colony formation was assessed by crystal violet staining in A549 cells after transfection. **C** Cell viability of A549 cells treated with MI-2 after 24 h was detected by CCK-8 assays. **D** The colony formation ability of A549 cells treated with MI-2 was performed by crystal violet staining. **E** The intensity of bioluminescence in orthotopic mouse model was detected by in vivo imaging system at 4 weeks after injection of A549-Luc cells. **F** Orthotopic lung cancer mice were sacrificed at 4 weeks after injection and the paraffin-embedded lung samples were performed on HE-staining. **G** H460 cells transfected with siMALT1_1 was used to established subcutaneous tumor growth in a mouse xenograft model. Growth curves of tumor volumes were determined every 2 days. **H** Representative IHC images of Ki67 in lung tissue specimens of orthotopic xenograft model. **I** Representative IHC images of MALT1 and Ki67 in tissue specimens of subcutaneous xenograft model. Each experiment was performed in triplicate and data are presented as mean ± SD. One-way ANOVA and Dunnett’s Multiple comparison test were used to analyze the data (*p < 0.05, **p < 0.01, ***p < 0.001)

The function of MALT1 in NSCLC cell malignancy was evaluated using a transient transfection model in A549 and H460 cells, the transfection was confirmed by immunoblotting (Fig. S1A). CCK-8 assays showed that downregulating MALT1 expression significantly inhibited cell proliferation (Fig. 1A and Fig. S1B). This finding was supported by colony formation assay results, which indicated that fewer colonies formed in the siMALT1 group (Fig. 1B and Fig. S1C). Conversely, overexpressed MALT1 promoted cell proliferation and the colony formation of NSCLC (Fig. 1A, B and Fig. S1B, C). MI-2, a small molecule inhibitor, which irreversibly inhibits the proteolytic activity of MALT1, reduced cell proliferation in a dose dependent manner (Fig. 1C and Fig. S1D). A lower concentration of MI-2 was used to assess colony formation capacity due to the small number of cells seeded at the beginning, and only 70–80% cells survived at 5 μM MI-2. Cell colony formation was also inhibited by MI-2 in a dose dependent manner (Fig. 1D and Fig. S1E). The above data demonstrate that cell proliferation in vitro is promoted by MALT1.

An orthotopic model was established by directly injecting A549-Luc cells into left lung of mice to confirm the oncogenic role of MALT1 in NSCLC. The mice were treated with DMSO or MI-2 (25 mg/kg) after 1 week and sacrificed at 4 weeks. The bioluminescence activity intensity in the NC group continuously increased, while that in the MI-2 group remained steady (Fig. 1E). HE staining revealed that more cells invaded alveoli and engulfed pulmonary vascular structures in NC group than in the MI-2 group (Fig. 1F). Lung tissue Ki67 IHC staining also demonstrated that MI-2 inhibited tumor growth (Fig. 1H). A subcutaneous tumor model confirmed that MALT1 knockdown considerably decreased tumor size and tumor growth compared with siNC treatment (Fig. 1G and Fig. S1F). The tumor tissues were removed to determine the expression of MALT1 and Ki67 by IHC (Fig. 1I and Fig. S1G). These findings collectively suggest that inhibition or MALT1 silence significantly suppresses tumor growth in vivo.

### Suppression of MALT1 inhibits NSCLC cell migration and radiation resistance

**Fig. 2:**
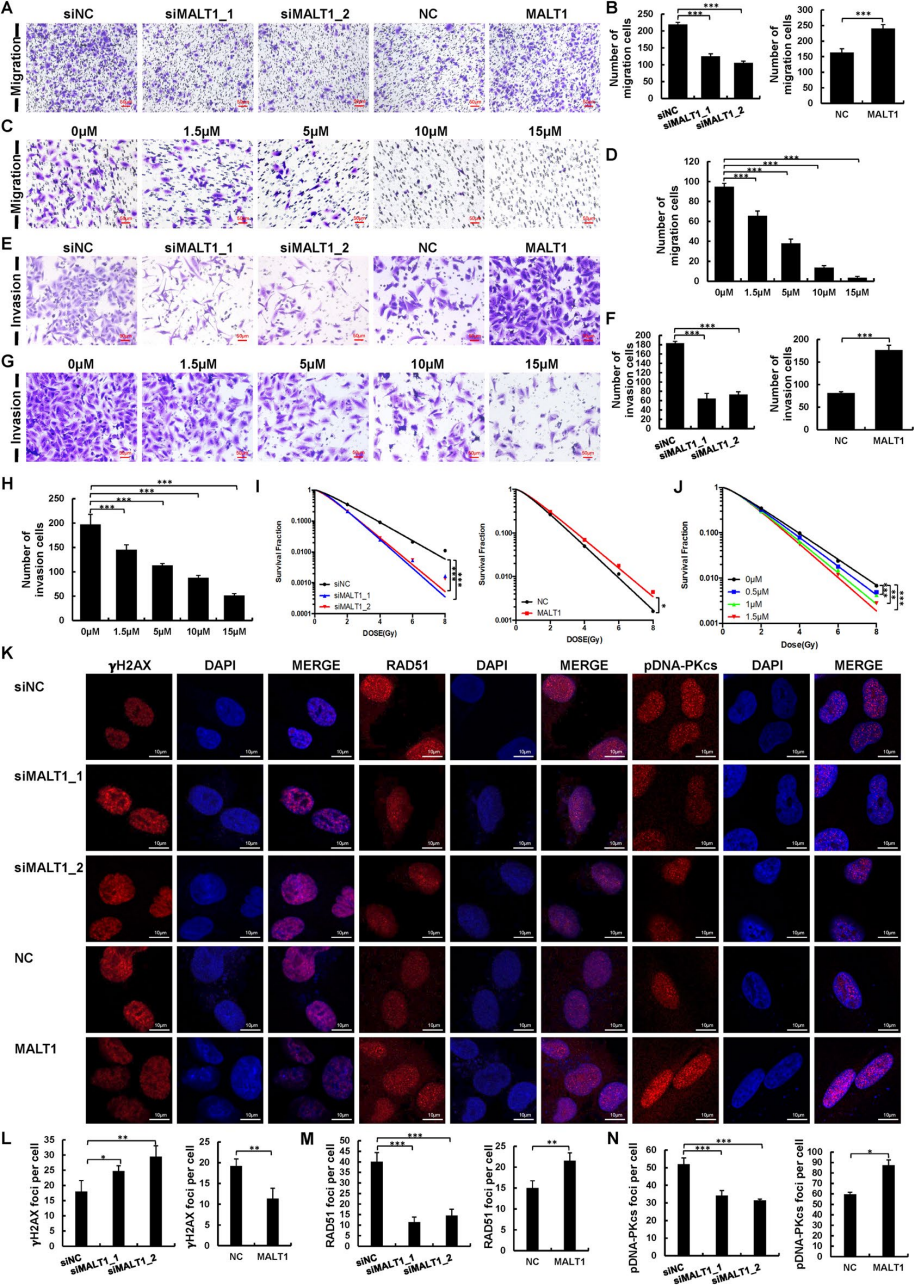
Suppression of MALT1 inhibited NSCLC cell migration, invasion and radiation resistance. **A, B** Cell migration of A549 cells transfected as described was determined by transwell assays. **C, D** After MI-2 treatment, A549 cell migration detected by transwell assays. **E, F** Cell invasion of A549 cells after transfection was performed by Matrigel invasiveness measurement. **G, H** The invasion ability of MI-2 treated A549 cells was detected by Matrigel invasiveness measurement. **I** The effects of MALT1 on A549 cells radiosensitivity after 0-8 Gy irradiation was determined through colony formation assays. **J** A549 cells were treated with 0-8 Gy irradiation, then immediately cultured with MI-2 for 24 h. **K-N** X-ray irradiated A549 cells were fixed 1 h later and γH2AX (**K**, **L**), RAD51 (**K**, **M**) and phosphorylated DNA-PKcs (**K**, **N**) foci formation was visualized by immunofluorescent staining. Each experiment was performed in triplicate and data are presented as mean ± SD. One-way ANOVA and Dunnett’s Multiple comparison test were used to analyze the data (*p < 0.05, **p < 0.01, ***p < 0.001)

Metastasis is a major hallmark of cancer and contributes to neoplastic progression. Transwell assays in A549 and H460 cells showed that MALT1 knockdown decreased cell mobility; more cells penetrated the membrane in siNC group than in the siMALT1 group (Fig. 2A, B and Fig. S2A, B). The same trend was observed in cells treated with MI-2 (Fig. 2C, D and Fig. S2C, D). Matrigel invasion was impaired cell invasion by MALT1 silencing (Fig. 2E, F and Fig. S2E, F) and by MI-2 inhibition in a dose dependent manner (Fig. 2G, H and Fig. S2G, H). In contrast, upregulation of MALT1 increased the cell migration and invasion in both A549 and H460 cells (Fig. 2A, B, E, F and Fig. S2A, B, E, F). Collectively, these results prove that MALT1 enhances the migration and invasion in NSCLC.

Given the association between radiation resistance and poor prognosis in NSCLC, and the regulation of DNA repair pathways by NF-κB dependent signaling (Aasland et al. 2019), we investigated the role of MALT1 in radiation response of NSCLC. Colony formation assays revealed that MALT1 knockdown decreased cell survival and enhanced radiosensitivity (Fig. 2I and Fig. S2I), as did MI-2 treatment, in a dose dependent manner (Fig. 2J and Fig. S2J). Immunofluorescence assays were performed to assess DNA damage repair in A549 and H460 cells. Results showed a significant increase in γ-H2AX foci after 8 Gy irradiation in the siMALT1 group compared to siNC group (Fig. 2K, L), and decreased expression of RAD51 and phosphorylated DNA-PKcs, the hallmark of homologous recombination (HR) and non-homologous end joining (NHEJ) pathway, respectively (Fig. 2K, M, N). Conversely, MALT1 overexpression suppressed cell radiosensitivity and γ-H2AX foci formation, but promoted the formation of RAD51 and phosphorylated DNA-PKcs foci in nucleus. Overall, MALT1 as upstream of NF-κB pathway enhanced NHEJ and HR pathways and decreased cell radiosensitivity in NSCLC suggesting that inhibition of MALT1 may be a novel strategy for NSCLC radiotherapy.

### CSN5 interacts with MALT1 to activate NF-κB signaling pathway

**Fig. 3:**
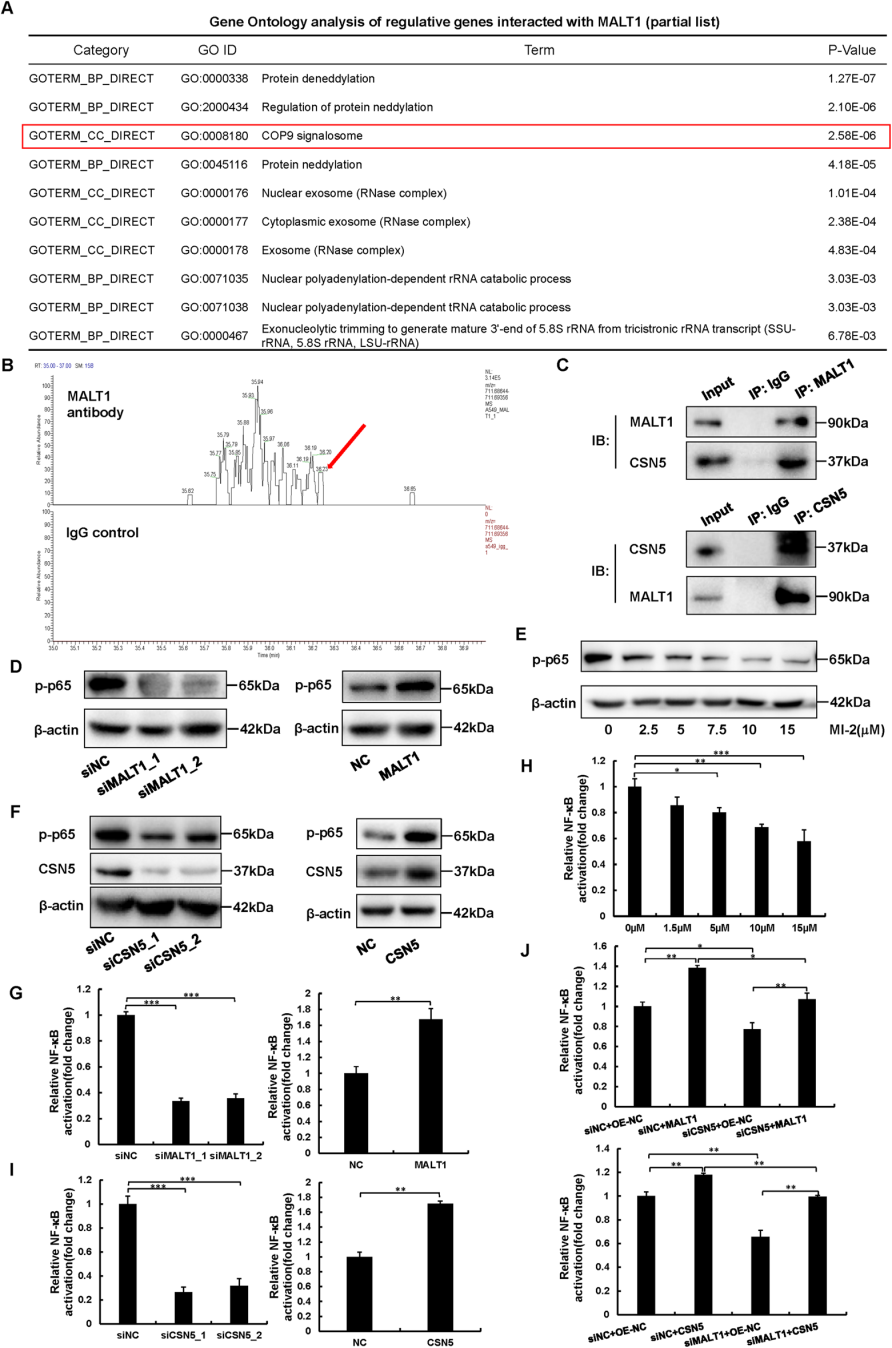
CSN5 interacted with MALT1 to activate NF-κB signaling pathway. **A** Gene Ontology categories by Gene Ontology analysis of genes interacted with MALT1 in A549 cells. BP biological process, MF molecular function, CC cellular component. The COP9 signalosome was highlighted. **B** In A549 cells, CSN5 protein in MALT1 Co-IP protein mix detected by MS, arrow indicated the identified CSN5 peptide peak. **C** The interaction between CSN5 and MALT1 in A549 cells was detected by Co-IP assays. **D-F** The effects of MALT1 (**D**), MI-2 (**E**) and CSN5 (**F**) on NF-κB signaling pathway activation in A549 cells were detected by immunoblotting. **G-H** Dual-luciferase reporter assays were used to analyze NF-κB activation in A549 cells after transfection (**G, I**) or MI-2 treatment (**H**). **J** Rescue assays were performed with dual-luciferase reporter assays to detected the NF-κB activation in A549 cells. Each experiment was performed in triplicate and data are presented as mean ± SD. One-way ANOVA, Dunnett’s Multiple comparison test and LSD multiple comparison test were used to analyze the data (*p < 0.05, **p < 0.01, ***p < 0.001)

LC–MS assays were performed to further explore the mechanism by which MALT1 promotes NSCLC malignant progression, and 930 proteins, including all eight substrates of the CSN complex, were identified. The top-ranked enriched Gene Ontology (GO) categories were “Protein deneddylation”, “Regulation of protein neddylation”, and “COP9 signalosome” according to the DAVID Bioinformatics Resources (https://david.ncifcrf.gov/) (Fig. 3A, B). The interaction between MALT1 and CSN5, the only one with catalytic active site in this complex (Lingaraju et al. 2014), was verified through Co-IP and immunoblotting (Fig. 3C and Fig. S3A). A549 and H460 cells were subjected to immunoblotting and dual-luciferase reporter assays to assess the contribution of CSN5 and MALT1 to NF-κB activation. The results showed that the protein level of phosphorylated p65, a hallmark of NF-κB activation, was reduced upon MALT1 or CSN5 downregulation and in dose-dependent manner after MI-2 treatment (Fig. 3D-F and Fig. S3B-D). Additionally, phosphorylated p65 was decreased in the MALT1 knockdown subcutaneous tumor model and MI-2 treated orthotopic tumor model (Fig. S3I, J). The luciferase activity of siMALT1 or siCSN5 was half that of siNC, and MI-2 also decreased the luciferase activity in a dose dependent manner (Fig. 3G-I and Fig. S3E-G). In contrast, overexpression of MALT1 or CSN5 strongly enhanced the protein level of phosphorylated p65 and the luciferase activity (Fig. 3D, F, G, I and Fig. S3B, D, E, G). Rescue assays confirmed that the increase in luciferase activity induced by exogenous MALT1 could be diminished by CSN5 knockdown, and co-transfection of CSN5 plasmid and MALT1 siRNA enhanced the luciferase activity compared to siMALT1 transfection alone (Fig. 3J and Fig. S3H). Due to the crucial role of protein neddylation in cancer, we determined the NEDD8 protein level in the whole cell lysates from MALT1 overexpression or CSN5 silenced NSCLC cells. As expected, the NEDD8 protein level was significantly increased after CSN5 silence, especially at the molecular-weight range of cullins (70-130 kDa). Upregulated MALT1 slightly decreased NEDD8 protein level and failed to decrease neddylation when CSN5 was downregulated, which suggested that interaction protein of MALT1 mediating protein neddylation was mainly the CSN (Fig. S3K, L). Overall, MALT1 activates NF-κB pathway via interacting with CSN5 in NSCLC.

### CSN5 mediates MALT1 protein stability

**Fig. 4:**
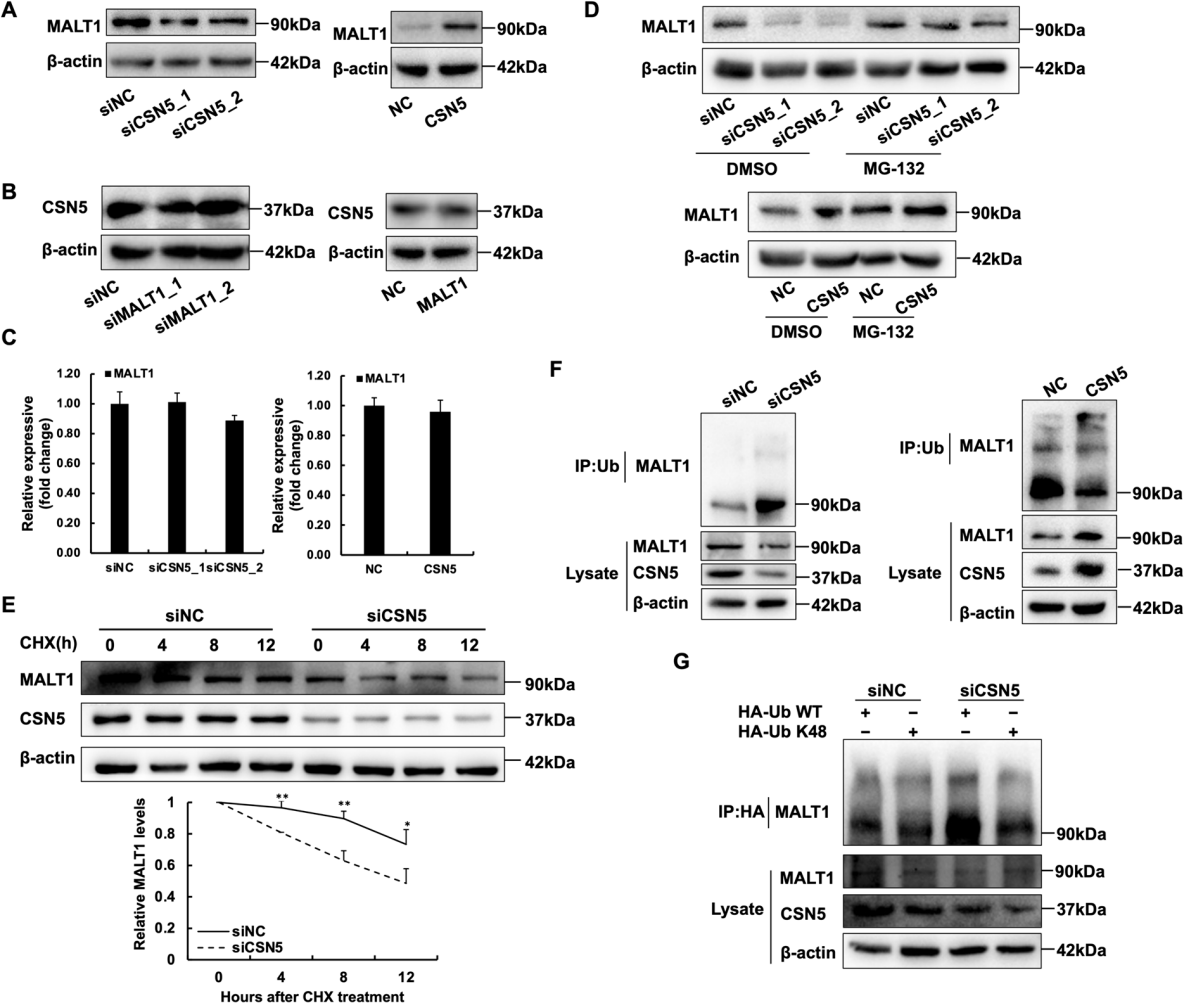
CSN5 mediated MALT1 protein stability. **A, B** The protein level of MALT1 (**A**) and CSN5 (**B**) in A549 cells after transfection was detected by immunoblotting. **C** The mRNA expression of MALT1 in A549 cells transfected as described was determined by qPCR. **D** 24 h after transfection, A549 cells were treated with MG-132 (50 μM) for 6 h, then the protein level of MALT1 was detected by immunoblotting. **E** CHX chase assays were conducted to measure MALT1 protein stability in CSN5 silenced A549 cells. Transfected A549 cells were treated with 10 μg/ml CHX for different times (4, 8, 12 h) and collected for immunoblotting. **F** Transfected A549 cells were analyzed by Co-IP with anti-polyubiquitin antibody for IP and anti-MALT1 antibody for immunoblotting. **G** CSN5 promoted MALT1 K48-linked poly-ubiquitination. HA-tagged Ub, K48 only mutant and CSN5 siRNA were co-transfected into A549 cells. Co-IP and immunoblotting were performed to detect the ubiquitination of MALT1. Each experiment was performed in triplicate and data are presented as mean ± SD. One-way ANOVA and Dunnett’s Multiple comparison test were used to analyze the data (*p < 0.05, **p < 0.01, ***p < 0.001)

Given the importance of CSN in regulating CRLs activation, we wondered whether CSN5 is required for MALT1 protein stability. Silencing CSN5 significantly decreased the protein level of MALT1. Overexpressed CSN5 increased MALT1 protein level, while MALT1 didn’t regulate the protein level of CSN5 (Fig. 4A, B and Fig. S4A, B). Further analysis demonstrated that CSN5 regulated MALT1 expression at the post-transcription level, as no changes were observed in MALT1 mRNA levels following CSN5 silence or overexpression (Fig. 4C and Fig. S4C, D). Transfected A549 and H460 cells were treated with MG-132, and the protein level of MALT1 was measured to confirm that CSN5 regulates MALT1 degradation. MG-132 almost completely restored MALT1 levels in the siCSN5 group to siNC levels without MG-132 treatment, and further increased MALT1 levels compared to those in group overexpressing CSN5 treated with DMSO (Fig. 4D and Fig. S4E). Moreover, CHX chase assays demonstrated that MALT1 protein was degraded more rapidly after CSN5 downregulation (Fig. 4E). These findings were further supported by immunoprecipitation experiments, which showed that CSN5 silencing increased MALT1 ubiquitination, while CSN5 overexpression decreased it (Fig. 4F and Fig. S4F). HA-tagged Ub wild type plasmids and HA-tagged Ub mutant plasmids in which all the lysine residues were mutated to arginine expect K48 (Ub K48 only) were conducted to further explore CSN5 mediated MALT1 poly-Ub chain linkage. And the result showed that the K48-linked ubiquitination level was increased as predicted but shown a significant weaken band compared to the overall ubiquitination after CSN5 silence (Fig. 4G and Fig. S4G). This suggested that CSN5 may not only mediated MALT1 protein stability but also mediated further activation. Taken together, these data indicated that CSN5 controls MALT1 stability via proteasomal pathway.

### FBXO3 is the E3 ligase involved in CSN5 mediated MALT1 stability

**Fig. 5:**
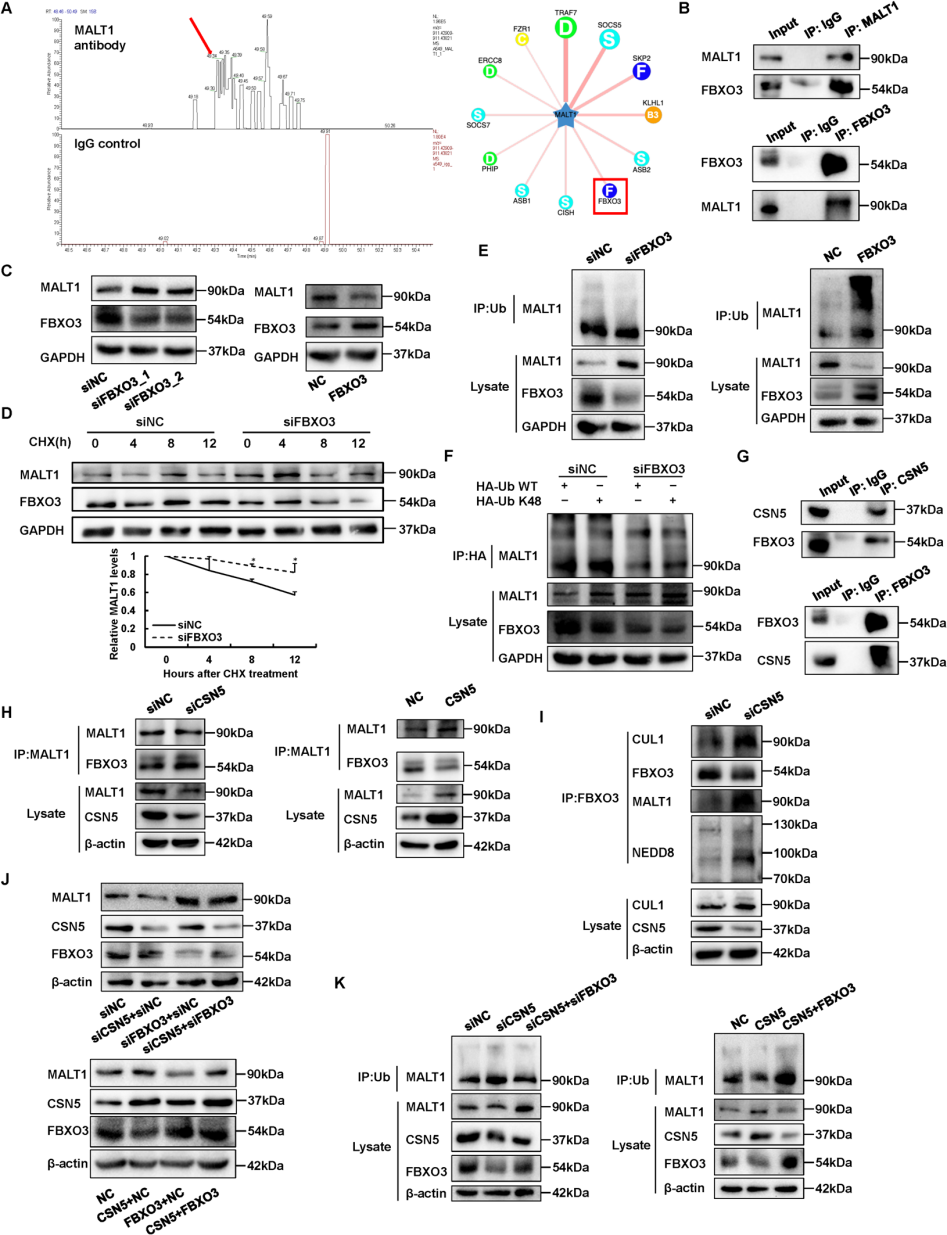
CSN5 mediated MALT1 stability through E3 ligase FBXO3. **A** In A549 cells, FBXO3 protein in MALT1 Co-IP protein mix detected by MS, arrow indicated the identified FBXO3 peptide peak. Potential E3 ligases of MALT1 were predicted by UbiBrowser. **B** The interaction between FBXO3 and MALT1 in A549 cells was detected by Co-IP assays. **C** The protein level of MALT1 in transfected A549 cells as described was detected by immunoblotting. **D** CHX chase assays were conducted to measure MALT1 protein stability in FBXO3 silenced A549 cells. Transfected A549 cells were treated with 10 μg/ml CHX for different times (4, 8, 12 h) and collected for immunoblotting. **E** Co-IP and immunoblotting assays determined the ubiquitination of MALT1 in transfected A549 cells. **F** FBXO3 promoted MALT1 K48-linked poly-ubiquitination. HA-tagged Ub WT plasmid or HA-tagged Ub K48 only plasmid and FBXO3 siRNA were co-transfected into A549 cells. Co-IP and immunoblotting were performed to detect the ubiquitination of MALT1. **G** The interaction between CSN5 and FBXO3 was confirmed using Co-IP assays in A549 cells. **H** Co-IP and immunoblotting assays determined the interaction between MALT1 and FBXO3 in transfected A549 cells. **I** CSN5 impaired the assembly of FBXO3 E3 ligase and the neddylation of CRL. Co-IP assays were performed to examine the protein binding to FBXO3 in A549 cells. **J** The siRNA or plasmid of CSN5 and FBXO3 were co-transfected into A549 cells and the MALT1 protein level was detected. **K** Co-IP and immunoblotting assays were performed to examine the ubiquitinated MALT1 in a rescue model of A549 cells. Each experiment was performed in triplicate. One-way ANOVA were used to analyze the data (*p < 0.05, **p < 0.01, ***p < 0.001)

CSN activation depends on E3 ligase; thus, we investigated the involvement of a specific CRLs to examine the mechanism by which CSN5 stabilizes MALT1. The LC–MS results and UbiBrowser (http://ubibrowser.bio-it.cn/ubibrowser/home/index) predicted FBXO3 to be a CRL of MALT1 (Fig. 5A). Subsequently, Co-IP and immunoblotting confirmed the interaction between MALT1 and FBXO3 in both A549 and H460 cells (Fig. 5B and Fig. S5A). Compared with that in the siNC group, the MALT1 protein level in the siFBXO3 treatment group was strongly enhanced, and upregulation of FBXO3 decreased MALT1 level compared to negative control (Fig. 5C and Fig. S5B). These findings also supported by CHX chase assays, which determined that MALT1 protein degradation was more slowly in siFBXO3 treated cells compared to siNC group (Fig. 5D). Moreover, FBXO3 knockdown reduced ubiquitination of MALT1 compared to siNC, and more ubiquitinated MALT1 was detected in FBXO3 overexpression group than control group (Fig. 5E and Fig. S5C). Also, the K48-linked ubiquitination level of MALT1 was decreased after FBXO3 silence and closely to the general poly-ubiquitination by HA-Ub WT immunoprecipitation, indicating that the E3 ligase FBXO3 poly-ubiquitinated MALT1 with K48 ubiquitin-chain and promoted its degradation (Fig. 5F and Fig. S5D). The interaction between CSN5 and FBXO3 was verified by co-IP assays to further test whether CSN5 regulates MALT1 degradation through FBXO3 (Fig. 5G and Fig. S5E). Moreover, there were more FBXO3 binding to MALT1 after CSN5 silence, and CSN5 overexpression impaired the interaction between MALT1 and FBXO3 (Fig. 5H and Fig. S5F). Besides, there were enhanced binding of MALT1 to FBXO3 after CSN5 downregulation, along with the upregulated neddylation of cullin1, which suggested that CSN5 enhances MALT1 stability by inhibiting deneddylation of FBXO3 specific CRLs. (Fig. 5I and Fig. S5G). Rescue experiments showed that decreased protein level of MALT1 by CSN5 silence was reversed by FBXO3 knockdown. However, MALT1 expression was enhanced by upregulated CSN5 which was reversed by overexpressed FBXO3 (Fig. 5J and Fig. S5H). Consistent with these findings, the increase in ubiquitination of MALT1 induced by siCSN5 was reduced by siFBXO3, and the increase in ubiquitination of MALT1 was restored by FBXO3 plasmid transfection (Fig. 5K and Fig. S5I). Furthermore, we confirmed that FBXO3 inhibited NF-κB activation by regulating MALT1 (Fig. S5J, K). These data confirmed that CSN5 regulates MALT1 stability via E3 ubiquitin ligase FBXO3.

### Overactivation of the CSN5/FBXO3/MALT1 regulatory *axis* is correlated with the poor prognosis in NSCLC patients

**Fig. 6:**
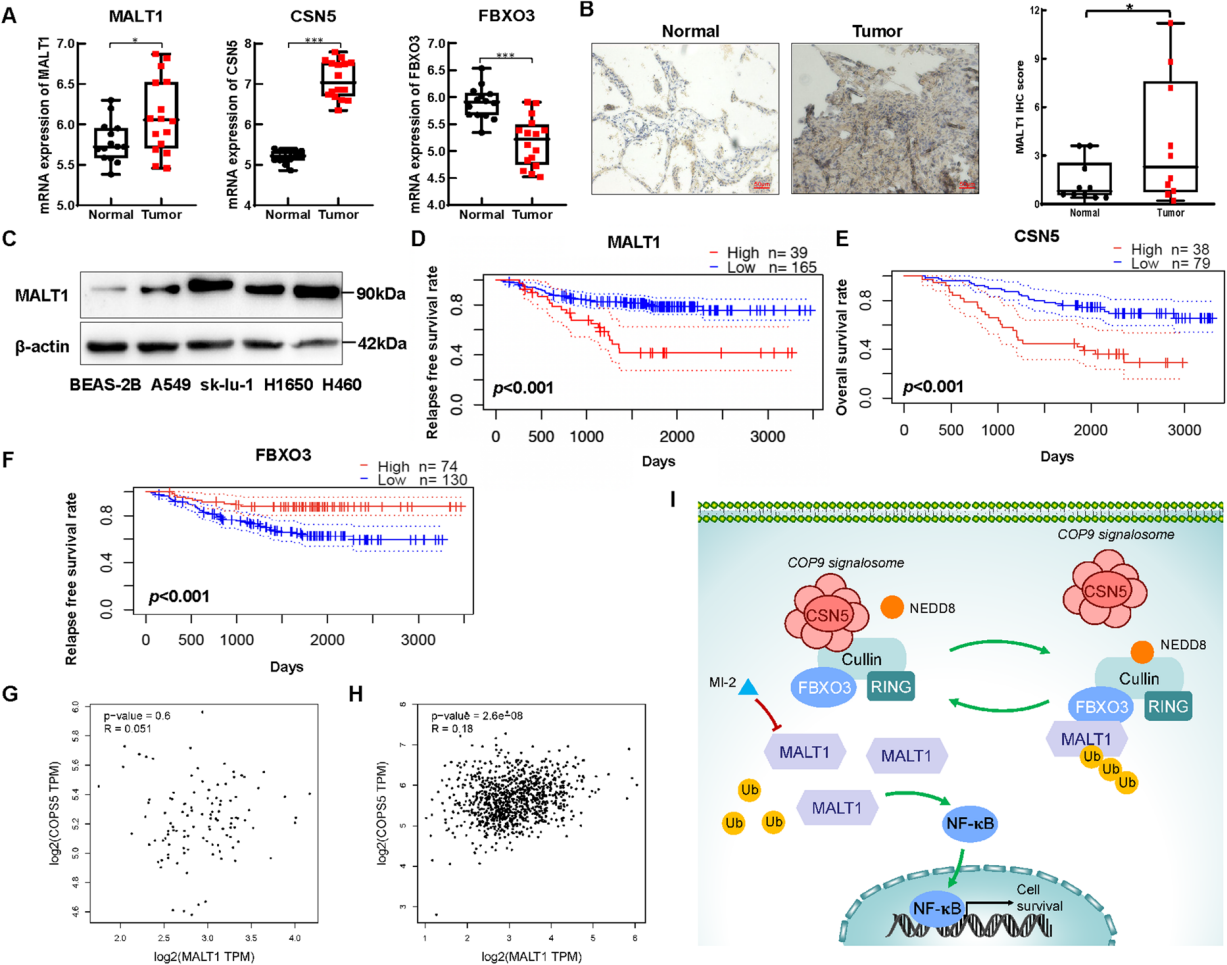
The over-activated CSN5/FBXO3/MALT1 regulatory axis is correlated with the poor prognosis of NSCLC patients. **A** The mRNA level of MALT1, CSN5 and FBXO3 in NSCLC tissues (n = 16) and normal lung tissues (n = 14) was analyzed by GEO database (GSE40275). **B** Representative IHC images of MALT1 in tissue specimens. Quantification of MALT1 levels according to IHC scores in right panel. **C** The protein level of MALT1 in bronchial epithelium cell BEAS-2B and NSCLC cell lines was determined by immunoblotting. **D** Relapse free survival Kaplan–Meier curves of NSCLC patients based on the expression of MALT1 were analyzed via PrognoScan database (GSE31210) and the cut-point value of MALT1 expression was 0.81. **E** Overall survival rate of NSCLC patients with high CSN5 expression was lower than those with low CSN5 expression. The data was analyzed by PrognoScan database (GSE13213) and the cut-point value of CSN5 expression was 0.68. **F** Relapse free survival Kaplan–Meier curves of NSCLC patients based on the expression of FBXO3 were analyzed via PrognoScan database (GSE31210) and the cut-point value of FBXO3 expression was 0.64. **G, H** The correlation between MALT1 and CSN5 in normal lung tissues (**G**) and NSCLC tissues (**H**), respectively. The data was analyzed by GEPIA website based on TCGA database. **I** Simplified model of over-activated CSN5/FBXO3/MALT1 regulatory axis promoting NSCLC malignancy via NF-κB pathway. In NSCLC, overexpressed CSN removes NEDD8 from Cullin-RING to inhibit E3 ligase FBXO3 activity resulting in reduction of MALT1 ubiquitination which contributes to MALT1 stabilization and keeps NF-κB activating, while small molecular inhibitor MI-2 irreversibly inhibits it. Each experiment was performed in triplicate and data are presented as mean ± SD. One-way ANOVA and Paired-Samples T test were used to analyze the data (*p < 0.05, **p < 0.01, ***p < 0.001)

To determine how CSN5/FBXO3/MALT1 regulatory axis functions in NSCLC, we first analyzed the expression of MALT1, CSN5 and FBXO3 in NSCLC tissues using the GEO database (GSE40275). The results showed higher mRNA expression of MALT1 and CSN5 in NSCLC tissues than in normal tissues, while FBXO3 mRNA expression was greater in normal tissues (Fig. 6A). Sequentially, IHC and immunoblotting assays confirmed that the protein level of MALT1 was greater in NSCLC tissues and cell lines than in adjacent normal tissues (*p* < 0.05) and in the bronchial epithelium cell line BEAS-2B (Fig. 6B, C). An analysis of GEO database (GSE31210 and GSE13213) revealed a lower relapse free survival (RFS) rate for patients with high MALT1 expression and a lower overall survival (OS) rate for patients with high CSN5 expression, while a higher RFS rate was observed for patients with high FBXO3 expression (Fig. 6D-F). Furthermore, MALT1 and CSN5 were found to be strongly correlated in NSCLC tumor tissues in the TCGA database, while no correlation was observed in normal lung tissues (Fig. 6G, H). Taken together, these findings indicate an association exists between CSN5/FBXO3/MALT1 regulatory axis and poor prognosis of NSCLC patients and that MALT1 is highly expressed in NSCLC.

## Discussion

MALT1, the only paracaspase in mammals, was identified with the critical role in immune system. Previous studies have demonstrated that MALT1 deficiency leads to infantile combined immunodeficiency and immune dysregulation due to the inability to produce memory and regulatory T cells (Punwani et al. 2015). In addition, MALT1 plays a vital role in lymphoma malignant progression, especially in diffuse large B-cell lymphoma (DLBCL), whose proliferation relied on CBM activating NF-κB pathway (Thome 2008). MALT1 has also been associated with radiotherapy resistance in MALT lymphoma patients, in which patients with MALT1 disruption had the best outcomes after radiation therapy (Mulligan et al. 2011). As for NSCLC, Pan et al. reported that MALT1 knockdown significantly impairs cell migration in vitro and in vivo (Pan et al. 2016). Our study revealed high MALT1 expression in NSCLC and a correlation with poor prognosis. Through functional studies, we verified that depletion of MALT1 hindered proliferation, migration, invasion, radiation resistance and tumor growth in vivo, confirming the oncogenic role of MALT1 in NSCLC. The proteolytic activity of MALT1 has attracted increasing attention as a potential drug target, and an ongoing clinical trial has tested its safety and efficacy for non-Hodgkin’s lymphoma (NHL) and chronic lymphocytic leukemia (CLL) and its potential use in the treatment of advanced or metastatic refractory solid tumors (Hamp et al. 2021). Here, our research provides compelling evidence for targeting MALT1 in NSCLC through MI-2 treatment, which suppresses of tumor malignancy.

In lymphocytes, MALT1 forming CBM complex facilitates TRAF6 oligomerization to polyubiquitinate IKKγ for NF-κB pathway activation (Sun et al. 2004). MALT1 can also directly interact with TRAF6 or form the cIAP2-MALT1 fusion protein to activate NF-κB pathway (Ho et al. 2005; Bardet et al. 2018). Proteolytic activity of MALT1 is key for prolonged and persistent NF-κB pathway activation via the cleavage of negative regulators of NF-κB pathway (Juilland and Thome 2018). Posttranslational modifications, especially ubiquitination, control MALT1 activity. Ubiquitination adds ubiquitin to a substrate protein and controls various biological processes, such as DNA repair, signal transduction and cell proliferation (Hershko and Ciechanover 1998). It has been verified that TRAF6 mediates K63-linked ubiquitination of MALT1 (Oeckinghaus et al. 2007), while HECTD3 promotes the formation of K27- and K29-linked polyubiquitin chains on MALT1, which does not mediate degradation but rather stabilizes it (Li et al. 2013; Cho et al. 2019). The monoubiquitination of MALT1 at Lys644 controls its protease function, which impacts the biological response of T cells (Pelzer et al. 2013; Schairer et al. 2020). However, the role of MALT1 ubiquitination in maintaining its stability is still poorly understood.

There are eight subunits in the CSN protein complex (designated as CSN1- CSN8), and CSN5 is the only subunit with CSN catalytic deneddylase activity (Gutierrez et al. 2020). So far, CRLs are the only confirmed substrates of CSN. CSN5 deactivates CRLs through removing NEDD8 from cullins to inhibit the ubiquitinated degradation of substrates by CRLs. Therefore, CSN can regulate all cellular biological processes, including NF-κB signaling, which is tightly regulated by ubiquitination (Q. Zhang et al. 2017). While CSN activating NF-κB may not be unique, deubiquitinylation of IκBα was facilitated by CSN2 to hamper NF-κB activation in HeLa cells (Schweitzer et al. 2007); however, NF-κB pathway activation was significantly impaired in CSN5 depleted thymocytes (Panattoni et al. 2008). Our study demonstrated that CSN5 activates NF-κB pathway in NSCLC through preventing the ubiquitin associated degradation of MALT1.

In our study, we first confirmed the presence of another E3 ligase FBXO3 on MALT1 through LC–MS assays. However, limited knowledge on its specific substrates of FBXO3, a member of the F-box family that belongs to the Skp-Cullin1-F box (SCF) superfamily of E3 ligases is available (Z. Zhang et al. 2021). It was previously demonstrated that FBXO3 mediates AIRE degradation (Shao et al. 2016), and promotes breast tumor migration through facilitating the ΔNp63α degradation (Niu et al. 2021). In this study, we first screened all the E3 ligases that bind to MALT1 through LC–MS, and identified CRLs among them. Only the FBXO3 was a CRL, which was also predicted by UbiBrowser. We confirmed that downregulated CSN5 enhanced the neddylation of FBXO3 E3 ligase and mediated more MALT1 binding to FBXO3, which resulted in the ubiquitinated degradation of MALT1. Moreover, CSN5 knockdown mediated ubiquitination of MALT1 was reversed by FBXO3 silence. These data suggested CSN5 stabilizes MALT1 via deactivating FBXO3.

In summary, our findings provide insight into the molecular basis of NSCLC progression through which CSN5 stabilizes MALT1 via E3 ligase FBXO3 to promote NSCLC malignant progression (Fig. 6I). Given these findings, the positive regulatory axis of CSN5/FBXO3/MALT1 confirmed in this study is a promising potential target for NSCLC therapy.

## Supplementary Information

Below is the link to the electronic supplementary material.

Supplementary file1 (PDF 4.19 MB)

## Data Availability

The data that supports the findings of this study are available on reasonable request from the corresponding author.
