# Supplementary material for: The COP9 signalosome stabilized MALT1 promotes Non-Small Cell Lung Cancer progression through activation of NF-κB pathway
Source: Cell Biol Toxicol. 2024 Jun 12;40(1):45. doi: 10.1007/s10565-024-09888-z (PMC11169058; doi:10.1007/s10565-024-09888-z)

Supplementary Information

The COP9 signalosome stabilized MALT1 promotes Non-Small Cell Lung Cancer progression through activation of NF-κB pathway

Yinghui Wang, Xuyi Deng, Jing Xie, Tianhao Lu, Rui Qian, Zhi Guo, Xin Zeng, Jing Liao, Zhenhua Ding, Meijuan Zhou* and Xinli Niu**

Supplementary Figure 1 MALT1 inhibition impaired proliferation of NSCLC cells. **A** The expression of MALT1 was detected in A549 or H460 cells transfected with siRNA or expression plasmid. **B** Cell proliferation of H460 cells was determined by CCK-8 assays at 24, 48, 72h after transfection, as described. **C** Colony formation was assessed by crystal violet staining in H460 cells after transfection. **D** Cell viability of H460 cells treated with MI-2 after 24h was detected by CCK-8 assays. **E** The colony formation ability of H460 cells treated with MI-2 was performed by crystal violet staining. **F** H460 cells transfected with siMALT1_2 was used to established subcutaneous tumor growth in a mouse xenograft model. Growth curves of tumor volumes were determined every 2 days. **G** Representative IHC images of MALT1 and Ki67 in tissue specimens of subcutaneous xenograft model. Each experiment was performed in triplicate and data are presented as mean ± SD. One-way ANOVA and Dunnett's Multiple comparison test were used to analyze the data (*p<0.05, **p<0.01, ***p<0.001).

Supplementary Figure 2 Suppression of MALT1 inhibited NSCLC cell migration, invasion and radiation resistance. **A, B** Cell migration of H460 cells transfected as described was determined by transwell assays. **C, D** After MI-2 treatment, H460 cell migration detected by transwell assays. **E, F** Cell invasion of H460 cells after transfection was performed by Matrigel invasiveness measurement. **G, H** The invasion ability of MI-2 treated H460 cells was detected by Matrigel invasiveness measurement. **I** The effects of MALT1 on H460 cells radiosensitivity after 0-8Gy irradiation was determined through colony formation assays. **J** H460 cells were treated with 0-8Gy

irradiation, then immediately cultured with MI-2 for 24h. Each experiment was performed in triplicate and data are presented as mean ± SD. One-way ANOVA and Dunnett's Multiple comparison test were used to analyze the data (*p<0.05, **p<0.01, ***p<0.001).

Supplementary Figure 3 CSN5 interacted with MALT1 to activate NF-κB signaling pathway. **A** The interaction between CSN5 and MALT1 in H460 cells was detected by Co-IP assays. **B-D** The effects of MALT1 (**B**), MI-2 (**C**) and CSN5 (**D**) on NF-κB signaling pathway activation in H460 cells were detected by immunoblotting. **E-G** Dual-luciferase reporter assays were used to analyze NF-κB activation in H460 cells after transfection or MI-2 treatment. **H** Rescue assays were performed with dual-luciferase reporter assays to detected the NF-κB activation in H460 cells. **I** Representative IHC images of phosphorylated p65 in tissue specimens of subcutaneous xenograft model. **J** Representative IHC images of phosphorylated p65 in lung tissue specimens of orthotopic xenograft model. **K, L** The NEDD8 protein level of whole cell lysates was determined by immunoblotting in transfected A549 (**K**) and H460 (**L**) cells. Each experiment was performed in triplicate and data are presented as mean ± SD. One-way ANOVA, Dunnett's Multiple comparison test and LSD multiple comparison test were used to analyze the data (*p<0.05, **p<0.01, ***p<0.001).

Supplementary Figure 4 CSN5 mediated MALT1 protein stability. **A, B** The protein level of MALT1 (**A**) and CSN5 (**B**) in H460 cells after transfection was detected by

immunoblotting. **C** The mRNA expression of CSN5 in A549 and H460 cells transfected as described was determined by qPCR. **D** The mRNA expression of MALT1 in H460 cells transfected as described was determined by qPCR. **E** 24h after transfection, H460 cells were treated with MG-132 for 6h, then the protein level of MALT1 was detected by immunoblotting. **F** Transfected H460 cells were analyzed by Co-IP with anti-polyubiquitin antibody for IP and anti-MALT1 antibody for immunoblotting. **G** CSN5 promoted MALT1 K48-linked poly-ubiquitination. HA-tagged Ub, K48 only mutant and CSN5 siRNA were co-transfected into H460 cells. Co-IP and immunoblotting were performed to detect the ubiquitination of MALT1. Each experiment was performed in triplicate and data are presented as mean ± SD. One-way ANOVA and Dunnett's Multiple comparison test were used to analyze the data (*p<0.05, **p<0.01, ***p<0.001).

Supplementary Figure 5 CSN5 mediated MALT1 stability through E3 ligase FBXO3. **A** The interaction between FBXO3 and MALT1 in H460 cells was detected by Co-IP assays. **B** The protein level of MALT1 in transfected H460 cells as described was detected by immunoblotting. **C** Co-IP and immunoblotting assays determined the ubiquitination of MALT1 in transfected H460 cells. **D** FBXO3 promoted MALT1 K48-linked poly-ubiquitination. HA-tagged Ub WT plasmid or HA-tagged Ub K48 only plasmid and FBXO3 siRNA were co-transfected into A549 cells. Co-IP and immunoblotting were performed to detect the ubiquitination of MALT1. **E** The interaction between CSN5 and FBXO3 was confirmed using Co-IP assays in H460 cells.

F Co-IP and immunoblotting assays determined the interaction between MALT1 and FBXO3 in transfected H460 cells. **G** CSN5 impaired the assembly of FBXO3 E3 ligase and the neddylation of CRL. Co-IP assays were performed to examine the protein binding to FBXO3 in A549 cells. **H** The siRNA or plasmid of CSN5 and FBXO3 were co-transfected into H460 cells and the MALT1 protein level was detected. **I** Co-IP and immunoblotting assays were performed to examine the ubiquitinated MALT1 in a rescue model of H460 cells. **J, K** Dual-luciferase reporter assays were used to analyze NF-κB activation in A549 (**J**) and H460 (**K**) cells after transfection. Each experiment was performed in triplicate. One-way ANOVA and LSD multiple comparison test were used to analyze the data (*p<0.05, **p<0.01, ***p<0.001).

Supplementary Table 1 siRNA and primer sequences list

Gene	Sequence
siMALT1_1	CTGTCGAGTTAATAACAAT
siMALT1_2	CCAATCTTGGATGCACTAA
siCSN5_1	GTACCAGACTATTCCACTT
siCSN5_2	GTCTCAGGTTATTAAGGAT
siFBXO3_1	GGGTGTCTATAGCCCGATT
siFBXO3_2	GACTATCGGGATCTAATCA
MALT1 primer	F 5’-TGGAAGCCCTATTCCTCACTACC-3’
	R 5’-CATGACACCAGTAGGTTCCTTGG-3’
CSN5 primer	F 5’-CCAGGAACCATTTGTAGCAGTGG-3’
	R 5’-GTCTGGTACTCAGAAGGTCCTTC-3’
GAPDH primer	F 5’-GGATATTGTTGCCATCAATGACC-3’
	R 5’-AGCCTTCTCCATGGTGGTGAAGA-3’

Supplementary Figure 1

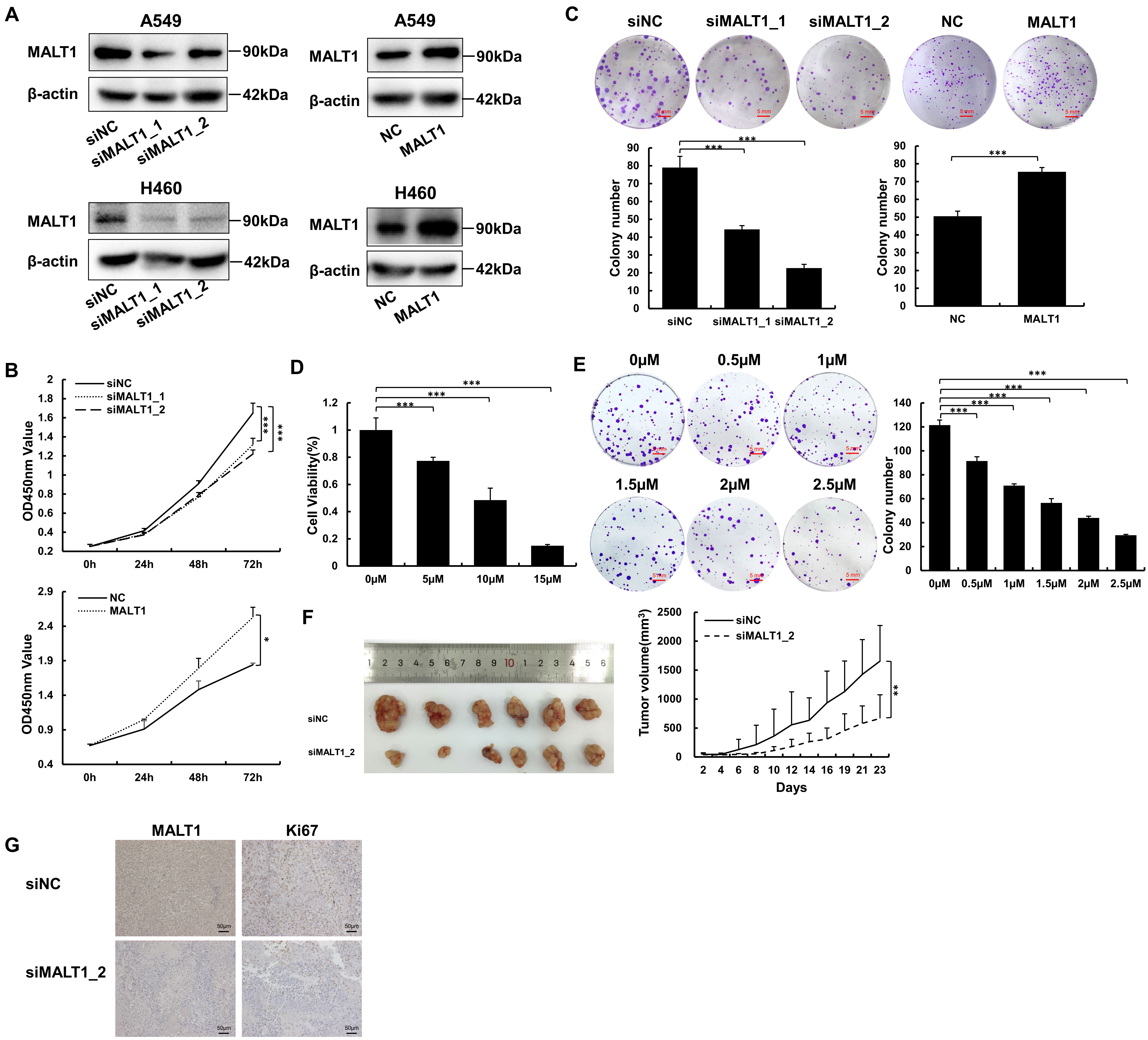

Supplementary Figure 2

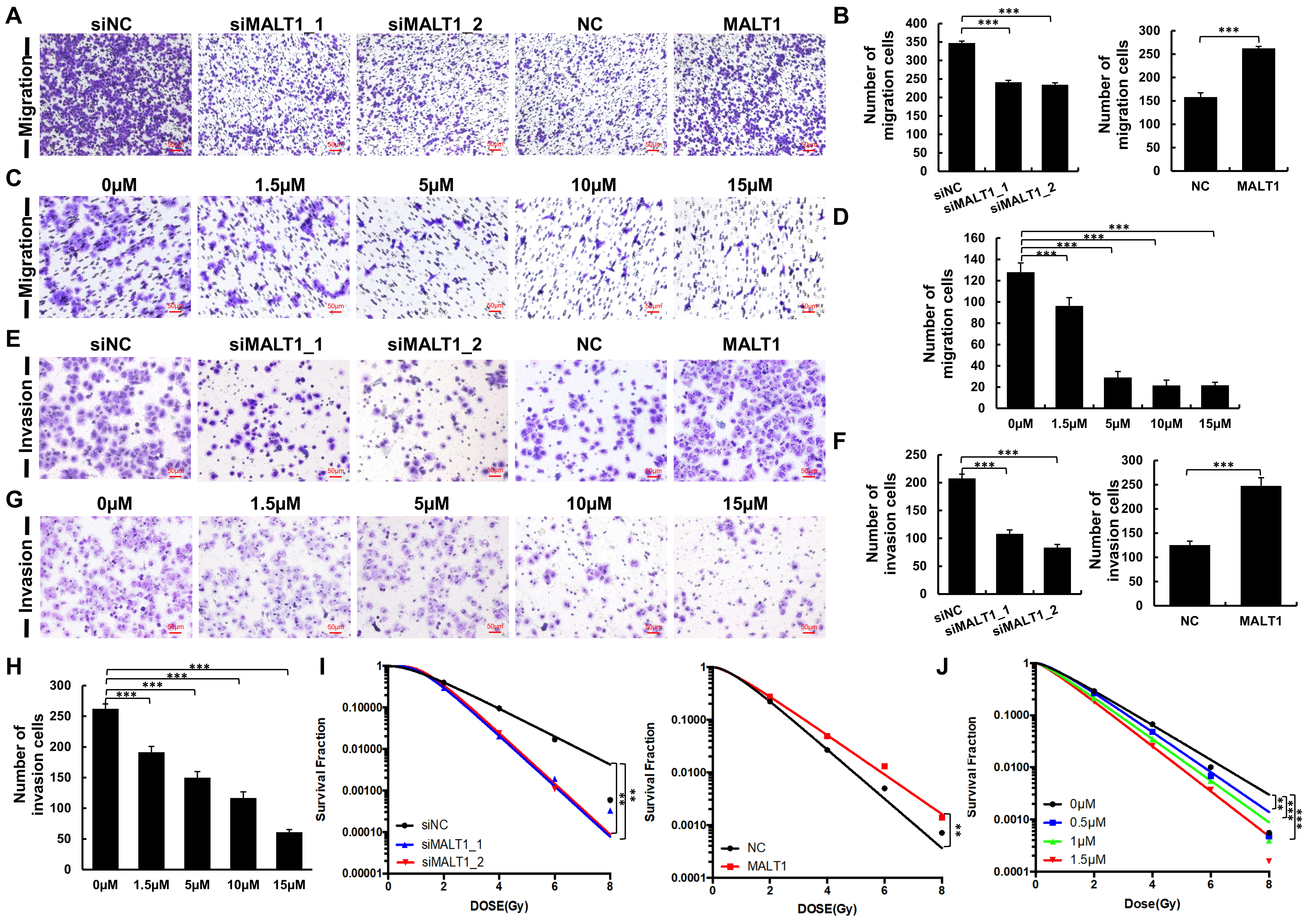

Supplementary Figure 3

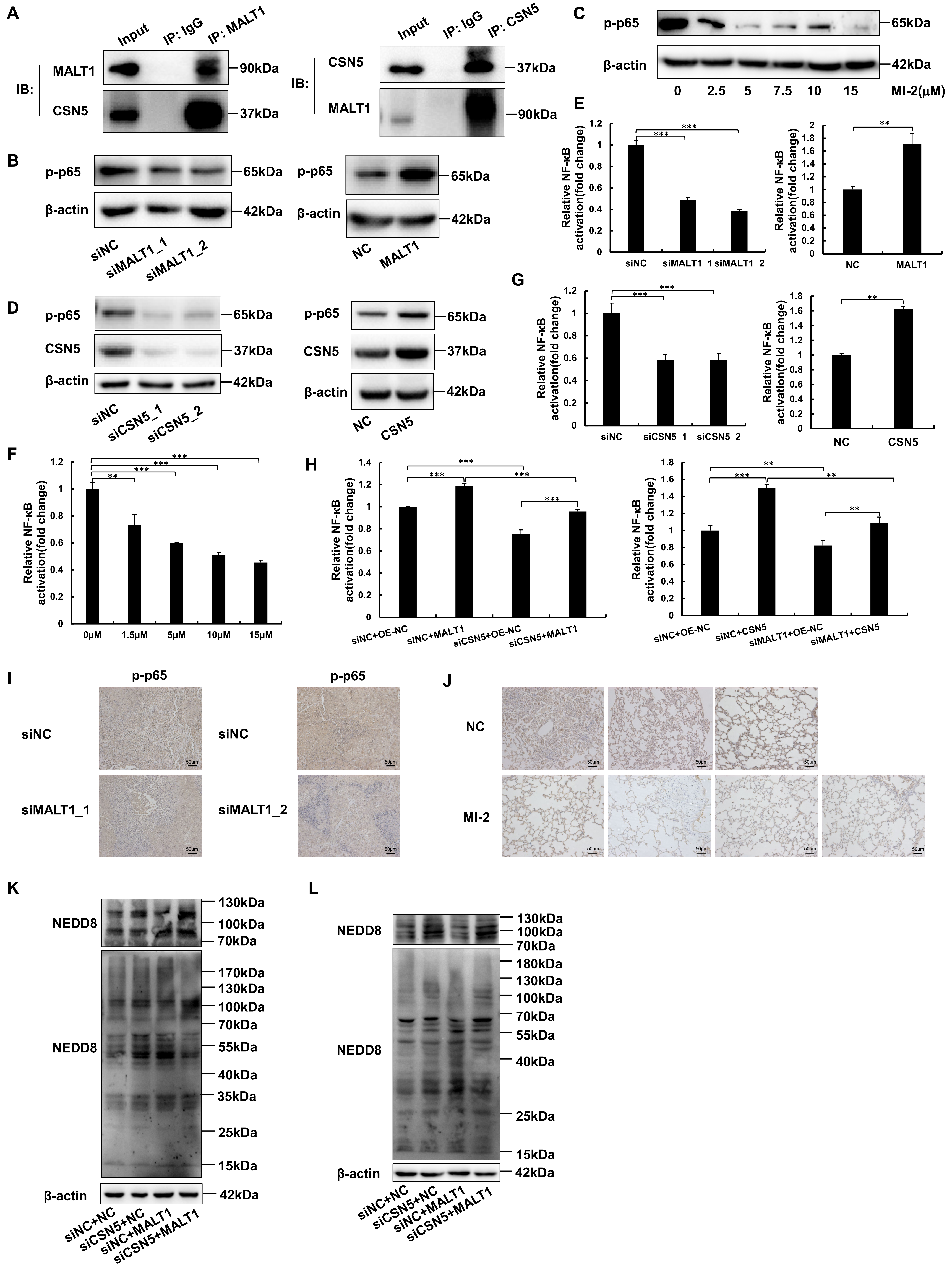

Supplementary Figure 4

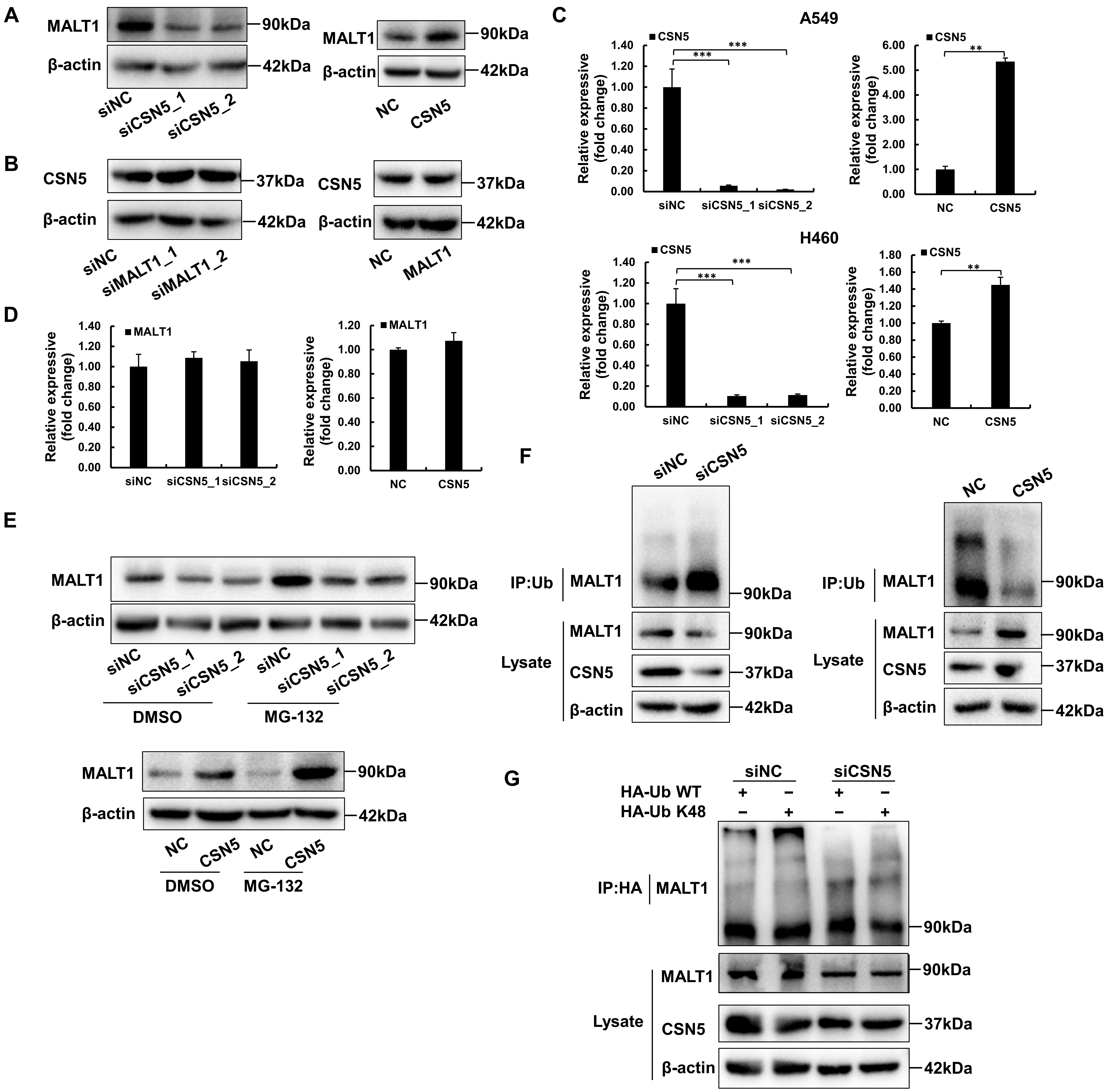

Supplementary Figure 5

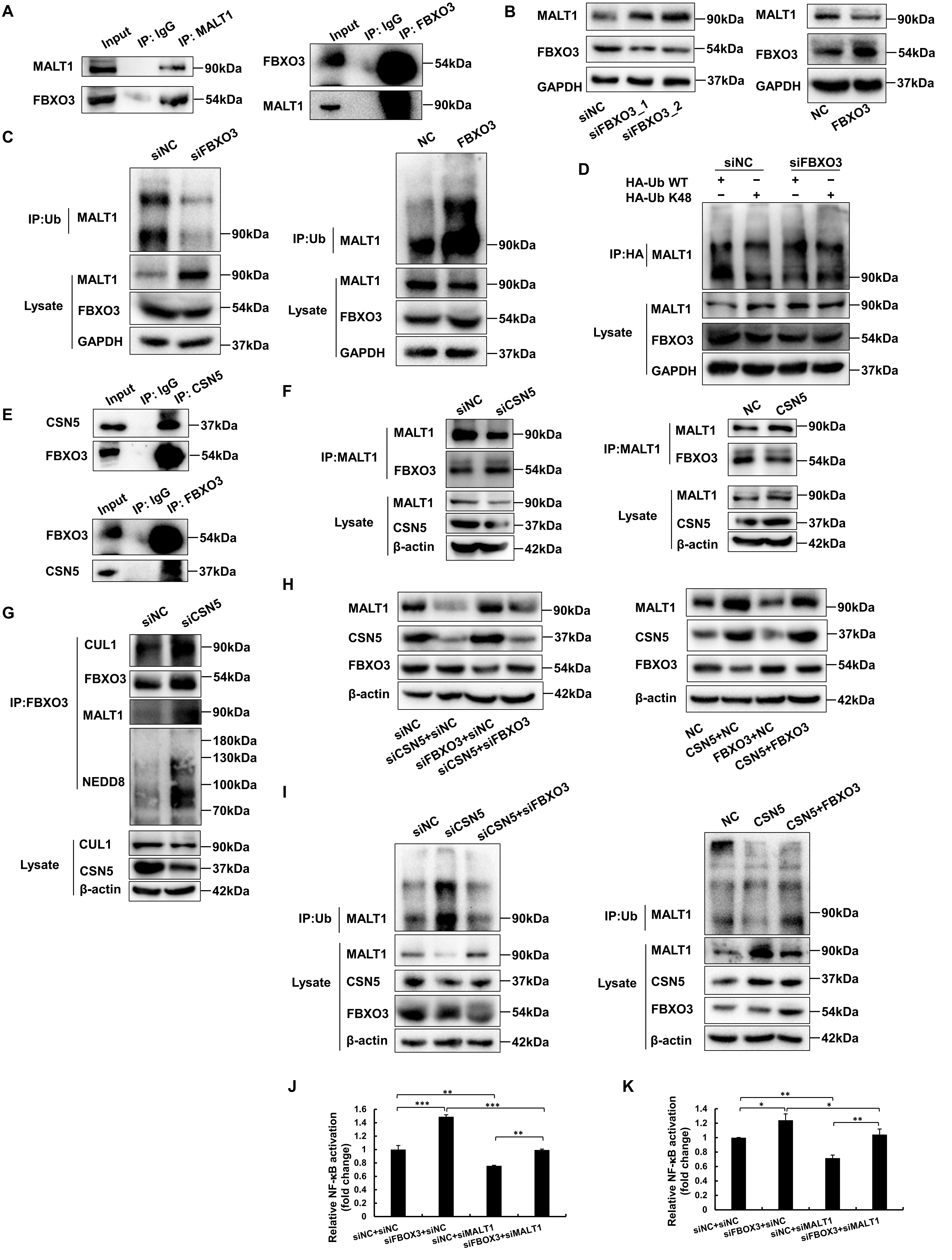

Supplement: Supplementary file 1 — Supplementary file1 (PDF 4.19 MB) [file 10565_2024_9888_MOESM1_ESM.pdf]
